# 5-Methylcytosine immunohistochemistry for predicting cutaneous melanoma prognosis

**DOI:** 10.1038/s41598-024-58011-z

**Published:** 2024-03-30

**Authors:** Jiraroch Meevassana, Shananya Varophas, Piyawan Prabsattru, Supitcha Kamolratanakul, Komkrit Ruangritchankul, Nakarin Kitkumthorn

**Affiliations:** 1 Center of Excellence in Burn and Wound Care, Faculty of Medicine, Chulalongkorn University, Bangkok, Thailand; 2 Department of Surgery, Division of Plastic and Reconstructive Surgery, Faculty of Medicine, Chulalongkorn University, Bangkok, Thailand; 3 Department of Clinical Tropical Medicine, Faculty of Tropical Medicine, Mahidol University, Bangkok, Thailand; 4 Department of Pathology, Faculty of Medicine, Chulalongkorn University, Bangkok, Thailand; 5 Department of Oral Biology, Faculty of Dentistry, Mahidol University, No. 6, Yothi Road, Ratchathewi District, Bangkok, 10400 Thailand

**Keywords:** Biological techniques, Cancer, Genetics, Molecular biology, Biomarkers, Molecular medicine

## Abstract

There is a correlation between DNA methylation and the diseased stage and poor survival. 5-methylcytosine (5-mC) is one of the epigenetic modifications of bases that researchers focus on. Staining with 5-mC immunohistochemistry was used to examine pathological samples taken from individuals diagnosed with cutaneous melanoma. Between Breslow levels 2 and 4, there was a significant difference in the H-score of 5-mC expression (p = 0.046). A significant reduction in 5-mC expression H-scores was seen in patients who were diagnosed with ulcers (p = 0.039). It was shown that patients with low 5-mC had a significantly worse overall survival rate (p = 0.027).

## Introduction

Cutaneous melanoma is an aggressive skin cancer with rapid metastatic potential and high mortality. Although it represents only 0.6% of all cancer cases, it is disproportionately responsible for an estimated 65% of skin cancer-related mortalities^1^. Unfortunately, the incidence of this cancer is increasing. Data from 2020 indicated that approximately 100,350 new cases were diagnosed and 6,850 deaths were attributed to cutaneous melanoma in the U.S., with predictions suggesting a continual rise^1^. At the cellular level, melanomas originate from unchecked melanocyte proliferation within the basal layer of the skin. These melanocytes are critical for melanin production and shield the skin from the damaging effects of ultraviolet (UV) rays^2^. However, when exposed to excessive UV light, the DNA within skin cells becomes vulnerable, leading to potential mutations. The pronounced genetic variability observed in cutaneous melanoma highlights its virulence and the need for effective therapeutic interventions^3^.

Genetic variability can be significantly influenced by epigenetics. Epigenetics is a process that can have a substantial impact on genetic diversity. It involves reversible alterations in gene expression, such as acetylation, methylation, and histone modification. These changes modify the phenotype without altering the genotype. This emphasises the significance of epigenetic mechanisms in influencing the range of observable traits and the development of diseases without altering the DNA sequence^4^. The addition of a methyl group to the 5^th^ carbon position of cytosine, resulting in the formation of 5-methylcytosine (5-mC), is one epigenetic type of DNA methylation, which plays a central role as a negative regulator of transcription initiation at CpG islands found in approximately 60% of genes and is involved in the maintenance of a constitutively inactivated, heterochromic genomic region by conferring genomic stability and integrity^4^. Global DNA hypomethylation, also known as genome-wide hypomethylation, can result in chromosomal abnormalities, proto-oncogene expression, and loss of transcriptional repression of the genome^5–7^.

Cancer genomes often exhibit global hypomethylation, leading to genomic instability, along with simultaneous hypermethylation of the promoter regions of specific tumour suppressor genes, resulting in their silencing^8,9^. This duality in methylation patterns presents both a challenge and an opportunity. The complexity of these patterns, intertwined with other epigenetic marks, adds layers of intricacy to the cancer epigenomic landscape^10,11^. These distinctive methylation signatures hold promise not only as diagnostic and prognostic markers but also as potential therapeutic targets.

Current research investigates how variations in DNA methylation in normal tissues may be used to create cancer biomarkers. Recent studies conducted at several research organizations have shown that human malignancies of the breast, liver, lung, pancreas, colon, prostate, and brain exhibit global hypomethylation, which is characterized by significantly decreased levels of 5-mC^12–14^. Despite the growing understanding that aberrant DNA methylation is an essential factor in the progression of melanoma, the epigenome of cutaneous melanoma is more hypomethylated than that of benign nevi^15^. This results in significantly reduced 5-mC levels, which is an epigenetic hallmark of melanoma. This process may be crucial for the epigenetic control of melanoma cell proliferation, growth, and advancement^16^. Such epigenetic alterations in melanoma can drive disease progression, affect metastatic potential, and modulate treatment responses. Global hypomethylation, a feature of many cancers, can lead to chromosomal instability and tumour progression, whereas hypermethylation of specific promoter regions can silence tumour suppressor genes and other protective pathways^8,9,17^. In melanoma, these methylation dynamics further interact with UV exposure, genetic mutations, and other epigenetic modifications, adding layers of complexity to the epigenomic landscape^10^.

We hypothesized that a reduction in 5-mC levels related to global methylation could be used to predict the prognosis and survival associated with the pathological characteristics of cutaneous melanoma and that this reduction might serve as a future targeted therapy option and prognostic marker.

## Results

### Clinicopathological characteristics

As shown in Table 1, a total of 48 patients with cutaneous melanoma were included in this study; 22 were males (45%), and 26 were females (55%). With a median age of 65, the patients' ages ranged from 28 to 94. Histological subtype analysis showed that 10.5% (five cases) of diagnoses were superficial spreading, 52% (25 cases) were nodular, and 37.5% (18 cases) were acral lentiginous. Most cases were at Breslow level 4, while Breslow levels 3, 2, and 1 made up 58%, 19%, 17%, and 6% of cases, respectively.

**Table 1:** Clinicopathological characteristics of patients with cutaneous melanoma and their association with the 5-mC H score.

Clinicopathological characteristics		n	H score of 5-mC expression		*p* value
			Median	Range	
Sex	Male	22 (45.8%)	69.47	1.85–162.05	0.482
	Female	26 (54.2%)	65.66	1.11–144.16	
Age (years)	≤ 65	25 (52.1%)	70.04	1.11–162.05	0.796
	> 65	23 (47.9%)	62.86	3.49–158.46	
Histological subtype	Superficial spreading	5 (10.4%)	62.86	10.02–158.46	0.262
	Nodular	25 (52.1%)	59.66	1.11–134.22	
	Acral lentiginous	18 (37.5%)	80.41	2.22–162.05	
Breslow level	1	3 (6.3%)	91.59	59.66–131.48	0.024*
	2	8 (16.7%)	113.81	20.35–162.05	
	3	9 (18.7%)	53.42	1.85–121.10	
	4	28 (58.3%)	61.41	1.11–158.46	
Tumour stage	Early stage (I-II)	21 (43.7%)	79.39	1.11–158.46	0.827
	Advanced stage (III-IV)	27 (56.3%)	62.86	1.85–162.05	
Ulcer	Presence	30 (62.5%)	55.09	1.11–158.46	0.039*
	Absence	18 (37.5%)	80.87	1.85–162.05	
Staging node	0	22 (45.8%)	65.41	1.11–158.64	0.881
	1	13 (27.1%)	70.04	4.72–162.05	
	2	6 (12.5%)	46.18	1.85–144.16	
	3	7 (14.6%)	59.66	20.35–128.13	
Recurrence^a^	Recurrent	29 (63.0%)	62.86	1.11–158.46	0.322
	Not recurrent	17 (37.0%)	79.31	2.22–162.05	
Death^a^	Dead	27 (57.5%)	68.45	1.11–158.46	0.949
	Alive	20 (42.5%)	64.43	2.22–162.05	
Total		48			

^a^Remarks for missing data.

The Kruskal‒Wallis test with post hoc pairwise comparison was used for the Breslow level and H score of 5-methylcytosine expression, and the results showed that 5-methylcytosine expression at Breslow levels 2 and 4 was significantly different (p = 0.046) and that the other parameters were not significantly different.

Spearman’s correlation for Breslow thickness and the H score of 5-methylcytosine expression resulted in rho = − 0.348 with statistical significance (p value = 0.015)*.

According to the AJCC Cancer Staging Manual, Eighth Edition, 27 out of 48 patients (56%) were diagnosed in the advanced stage (III–IV) and 21 (44%) were in the early stage (I–II). Out of the total, 30 individuals (62.5%) had ulcers, while 18 individuals (37.5%) did not.

Regarding the nodal stage, most patients were at stage 0 (46%), with a minority at stage 1 (27%), 2 (12.5%), and 3 (14.5%). The median duration for disease-free survival (DFS) follow-up was 13 months (range: 3–52 months), while the median period for overall survival (OS) follow-up was 25 months (range: 4–95 months). The calculated H scores ranged from 1.11 to 162.05, with a median score of 68.68.

### 5-mC H score and clinicopathological correlation

**Figure 1:**
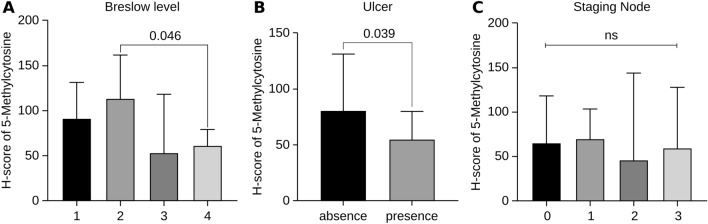
Comparison of 5-mC H scores in subgroups based on (**a**) The H score of 5-mC expression differed significantly between patients with Breslow levels 2 and 4 (p = 0.046), with no significant differences observed in the other scores. (**b**) Patients with ulcers showed significantly lower H scores for 5-mC expression (p = 0.039) compared to those without ulcers. (**c**) In patients with stages 2 and 3 disease, the metastatic lymph nodes showed lower 5-mC H scores compared to patients with stages 0 and 1 disease, but this difference was not statistically significant (p = 0.881).

The Kruskal‒Wallis test with post hoc pairwise comparison was used to analyse the correlation of the H score of 5-mC expression with each clinicopathological characteristic. The results revealed that the H score of 5-mC expression was significantly different between patients with Breslow levels 2 and 4 (p = 0.046; Fig. 1a), while the other scores were not significantly different. Patients who presented with ulcers had significantly lower H scores for 5-mC expression (p = 0.039; Fig. 1b) than those without ulcers (Table 1). The metastatic lymph nodes in patients with stages 2 and 3 disease had decreased 5-mC H scores than those in patients with stages 0 and 1 disease; however, this difference was not significant (Fig. 1c). In addition, there was no association between the H score of 5-mC expression and other factors, including recurrence (p = 0.322) and death (p = 0.949).

### 5-mC H score and outcome analysis

**Figure 2:**
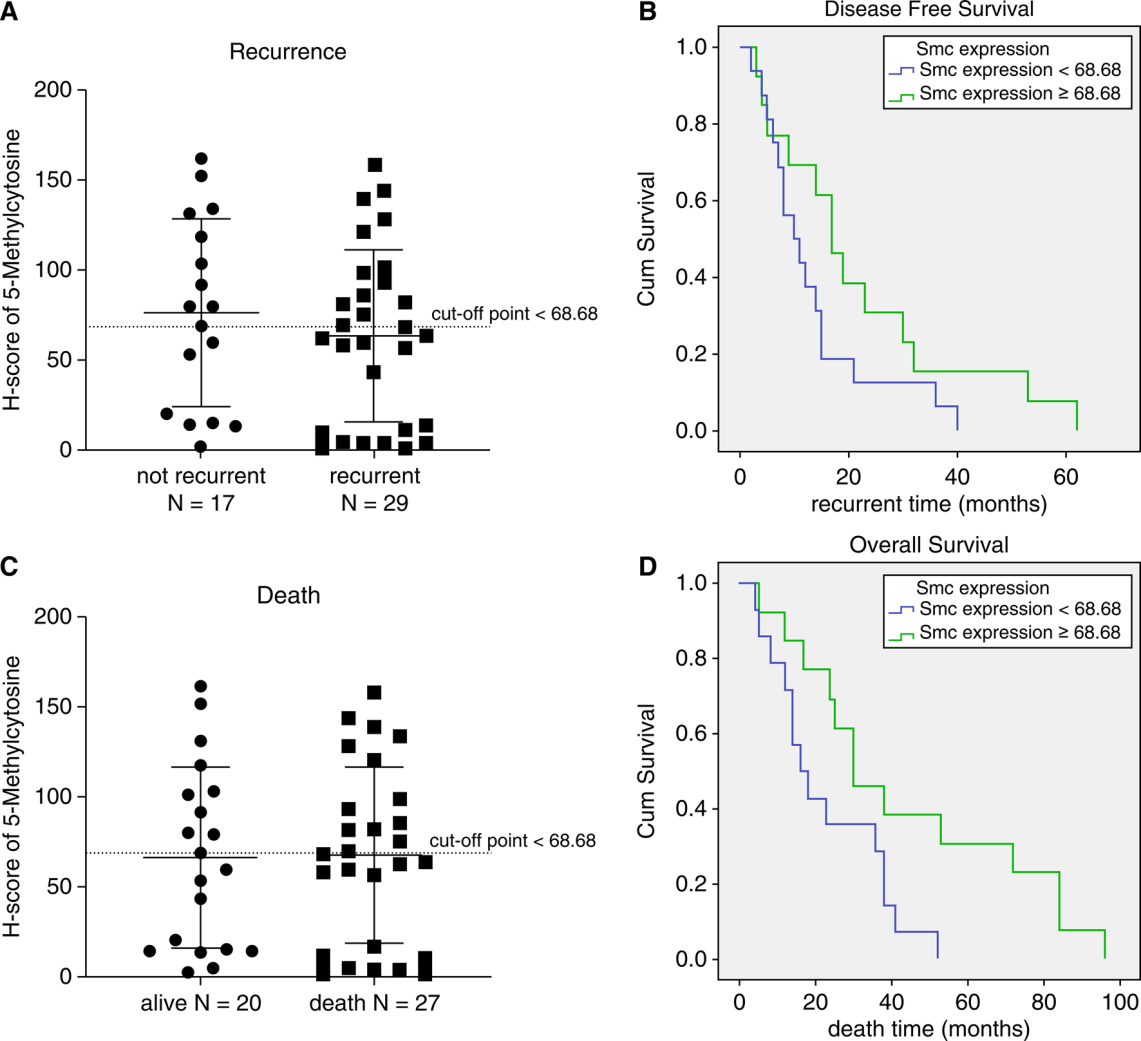
Prognosis prediction using the 5-mC H score. A median H score of 68.8 was utilized as the cut-off. (**a**) Comparison between the patients with recurrence and nonrecurrence. (**b**) Kaplan–Meier curve showing that patients with an H score less than 68.68 had a shorter disease-free survival time than patients with 5-mC expression scores of ≥ 68.68; however, the difference was not significant (p = 0.123). (**c**) Comparison between dead and surviving patients. (**d**) Kaplan–Meier curve showing that patients with an H score less than 68.68 had a shorter overall survival time (p = 0.037).

The median DFS and OS in this study were 17 months (range 3–62 months) and 30 months (range 5–96 months), respectively. We used the median H score (68.68) as the cut-off, as shown in Fig. 2a and c. Patients with 5-mC expression scores of ˂ 68.68 had shorter DFS than patients with 5-mC expression scores of ≥ 68.68; however, the difference was not significant (p = 0.123; Table 2 and Fig. 2b). In contrast, mortality outcomes were significantly predicted by 5-mC H scores of ≥ 68.68 (p = 0.037) and Breslow levels 3 (p = 0.040) and 4 (p = 0.037; Table 3). Multivariate analysis also demonstrated that a 5-mC expression score of ≥ 68.68 was an independent variable for OS (HR = 0.293, p = 0.025; Table 3 and Fig. 2d) in patients with cutaneous melanoma.

**Table 2:** Univariate Cox regression analysis for recurrence based on the 5-mC H score in patients with cutaneous melanoma.

Characteristics		Recur (N, %)	Univariate analysis		
			HR	95% CI	*p* value
Sex	Male	11 (22.9%)	1		
	Female	18 (37.5%)	1.376	0.631–3.001	0.421
Age (years)	≤ 65	13 (27.1%)	1		
	> 65	16 (33.3%)	1.016	0.478–2.161	0.967
Histological subtype	Superficial spreading	4 (8.3%)	1		
	Nodular	17 (35.4%)	0.723	0.238–2.197	0.567
	Acral lentiginous	8 (16.7%)	0.531	0.154–1.834	0.317
5-methylcytosine	H-score < 68.68	16 (33.3%)	1		
	H-score ≥ 68.68	13 (27.1%)	0.540	0.247–1.181	0.123
Breslow level	1	1 (2.1%)	1		
	2	3 (6.25%)	1.000	0.000–49,485.233	1.000
	3	5 (10.4%)	1.000	0.000–47,283.038	1.000
	4	20 (41.7%)	1.000	0.000–46,352.926	1.000
Tumour stage	Early stage (I-II)	9 (18.8%)	1		
	Advanced stage (III-IV)	20 (41.7%)	1.019	0.458–2.265	0.964
Ulcer	Absence	8 (16.7%)	1		
	Presence	21 (43.8%)	1.067	0.467–2.438	0.879
Staging node	0	11 (22.9%)	1		
	1	8 (16.7%)	0.931	0.356–2.438	0.884
	2	5 (10.4%)	0.831	0.283–2.443	0.737
	3	5 (10.4%)	2.312	0.771–6.940	0.135
	Total	29 (60.4%)

**Table 3:** Univariate and multivariate Cox regression analyses for mortality based on the 5-mC H score in patients with cutaneous melanoma.

Characteristics		Death (N, %)	Univariate analysis			Multivariate analysis		
			HR	95% CI	*p* value	HR	95% CI	*p* value
Sex	Male	10 (20.8%)	1					
	Female	17 (35.4%)	1.209	0.543–2.692	0.643			
Age (years)	≤ 65	13 (27.1%)	1					
	> 65	14 (29.2%)	0.539	0.247–1.174	0.120			
Histological subtype	Superficial spreading	4 (8.3%)	1					
	Nodular	16 (33.3%)	0.960	0.308–2.988	0.944			
	Acral lentiginous	7 (14.6%)	0.637	0.177–2.291	0.489			
5-methylcytosine	H-score < 68.68	14 (29.2%)	1			1		
	H-score ≥ 68.68	13 (27.1%)	0.391	0.162–0.946	0.037*	0.293	0.100–0.857	0.025*
Breslow level	1	1 (2.1%)	1			1		
	2	4 (8.3%)	0.102	0.008–1.314	0.080	0.304	0.020–4.680	0.393
	3	5 (10.4%)	0.070	0.006–0.880	0.040*	0.103	0.008–1.306	0.079
	4	17 (35.4%)	0.076	0.007–0.856	0.037*	0.112	0.010–1.272	0.077
Tumour stage	Early stage (I–II)	9 (18.8%)	1					
	Advanced stage (III–IV)	18 (37.5%)	1.609	0.687–3.765	0.273			
Ulcer	Absence	9 (18.8%)	1					
	Presence	18 (37.5%)	1.362	0.588–3.157	0.471			
Staging node	0	11 (22.9%)	1					
	1	5 (10.4%)	1.155	0.393–3.393	0.793			
	2	6 (12.5%)	1.613	0.569–4.576	0.369			
	3	5 (10.4%)	1.801	0.589–5.510	0.302			
	Total	27 (56.3%)						

## Discussion

Global hypomethylation in cutaneous melanoma has been reported to be associated with genomic instability, activation of oncogenes, and altered immune responses. This extensive reduction in methyl groups, primarily from repetitive sequences, can lead to chromosomal abnormalities, contributing to tumour progression^18,19^.

In this study, the severity of cutaneous melanoma correlated with the H score. For example, the Breslow level and ulceration were significantly associated with the H score (p values of 0.024 and 0.039, respectively). Based on these findings, we conclude that hypomethylation is associated with the aggressiveness of cutaneous melanoma. The recurrence and death rates in this study were 60.4% and 56.3%, respectively (Tables 2 and 3), and there was no correlation between any of the other factors, including sex, age, histological subtype, Breslow level, tumour stage, ulcer status, or staging node, except for the H score, which indicated a trend towards a higher recurrence rate for H scores < 68.68 than for H scores > 68.68. The recurrence rate can be predicted based on global hypomethylation by evaluating 5-mC levels and H scores.

No correlations were found between factors such as sex, age, histologic subtype, tumour stage, ulcer status, or nodal stage and mortality. However, the H score and Breslow level indicated a higher death rate when the H score was less than 68.68 (HR 0.391, p = 0.037). In addition, multivariate analysis revealed that the mortality rate was significantly associated with the H score at the cut-off point of 68.68 (p = 0.025). We also discovered that hypomethylation was associated with high Breslow levels, with Breslow levels 3 and 4 having HRs of 0.07 and 0.076, respectively, and p values of 0.04 and 0.037, respectively. In essence, global hypomethylation measured by the H score from 5-mC IHC offers a promising avenue for predicting both recurrence and mortality in patients with cutaneous melanoma. Our study robustly established that 5-mC IHC can serve as a powerful tool for mortality prediction, potentially refining patient prognosis and enhancing personalized treatment strategies.

The evolving understanding of cutaneous melanoma severity and its intricate association with hypomethylation has taken centre stage in oncology research. In this study, elucidation of the H score as an instrumental predictive marker has established a compelling avenue for further exploration. Melanoma exhibits global hypomethylation, which plays an important role in predicting patient prognosis^15,20,21^.

Our findings are consistent with those of Ehrlich^22^, who emphasized the connection between hypomethylation and various types of cancer. This finding suggested a broad and significant role of epigenetic modulation in tumorigenesis^22^. Furthermore, while Cohen et al.^23^ proposed the initial concept of the H score, its application was limited to breast cancer, highlighting the innovative nature of our melanoma-centric focus. In contrast, Hyams et al.^24^ emphasized that age, sex, and histological subtype were potential prognostic factors. The discrepancy between our results and theirs emphasizes the complex character of melanoma and thus begs for a more nuanced understanding, perhaps by merging different datasets.

The utility of the Breslow level, as emphasized by both Atique et al.^25^ and Rashed et al.^26^, has universal acceptance in determining the prognosis of melanoma. Interestingly, Ecsedi et al.^27^ suggested a correlation between the Breslow depth and hypomethylation, and our investigation thoroughly confirmed this hypothesis. Micevic et al.^16^ provided basic knowledge on the potential of the 5-mC score among molecular markers. Our research extends this narrative by emphasizing the predictive process of the H score. Similarly, the preliminary work of Yang et al.^28^ on molecular markers laid the groundwork for our research. Fath et al.^29^ suggested that epigenetic factors may have a greater influence on melanoma than genetic alterations, and our data indirectly support this view. This evolving understanding is reinforced by the work of Van Doorn et al.^30^, who revealed an association between DNA methylation and the aggressiveness of tumours in various skin cancers. Constrained promoter hypermethylation and global hypomethylation are epigenetic characteristics of melanoma, and both have an impact on tumour behaviour^31^.

There are many DNA methylation analysis techniques, including methylation-specific polymerase chain reaction (PCR), pyrosequencing, and bisulfite genome sequencing^32,33^. IHC is unique and provides contextual awareness. The use of IHC in this study was crucial for studying methylation in tissue architecture^34^. IHC is enhanced by spatial resolution and is different from other methods because it shows both "what" and "where." This localization capacity is useful for studying methylation during tissue- and cell-specific events^35,36^. Determining whether tumour cells exhibit methylation alterations can reveal disease progression and guide targeted therapy^37^. This technique is simple and affordable, making it a suitable choice for many laboratories. This practicality and fast turnaround enable prompt clinical testing. The versatility of IHC for evaluating paraffin-embedded and frozen tissue slices allows important historical materials to be studied in addition to new samples^38,39^. This study is limited by the small number of patients who were analysed. A multicenter study, or one that includes a greater number of participants, will produce more data and have better value. Furthermore, investigating the molecular mechanisms related to the connections between 5-mC and melanoma is essential. For confounding factors such as age, tumour location, and treatment history. Given that the studies were carried out at an average age of 65, the patients' ages varied from 28 to 94 years. Some research implies that ageing can affect methylation levels^40,41^. In this study, there was no significant difference in age groups among the majority of patients. According to the literature review, there is no difference in methylation between methylation level and location^31,42,43^. Some papers found that previous therapy had no effect on methylation levels^31,44^, and in our analysis, we recruited a melanoma patient who had not previously received treatment. Finally, the study's results were unaffected by age, tumour location, or treatment experience.

Our results confirmed the potential of hypomethylation as an indicator of cutaneous melanoma severity. Incorporating such epigenetic markers, particularly the H score, into routine clinical assessments could refine the prognostic and treatment strategies for patients, ultimately leading to more personalized and effective care. The challenges posed by these complex methylation patterns in melanomas are significant. However, distinctive methylation signatures in melanoma hold promise as diagnostic, prognostic, and therapeutic markers. The development of technologies such as whole-genome bisulfite sequencing and innovations such as CRISPR/Cas9 epigenetic editing provides new avenues for exploring, understanding, and targeting the methylation landscape in cutaneous melanoma and other cancers. Considering the potential benefits, combining the conversion of global hypomethylation to hypermethylation in melanoma with other targeted therapies like molecularly targeted tyrosine kinase inhibitors (TKIs), BRAF, MEK, etc. could enhance treatment efficacy for patients. Therefore, our results showed that 5-mC IHC can be used as an alternative prognostic marker for cutaneous melanoma. This technique is inexpensive and accurate, and errors are reduced by using automation to calculate the H score.

### Summary points

1. Global DNA hypomethylation, also known as genome-wide hypomethylation, can result in chromosomal abnormalities, proto-oncogene expression, and loss of transcriptional repression of the genome.
2. Melanoma exhibits global hypomethylation within the whole genome, which plays an important role in predicting patient prognosis.
3. Our study robustly established that 5-mC IHC can serve as a powerful tool for mortality prediction, potentially refining patient prognosis and enhancing personalized treatment strategies.
4. The elucidation of the H score as an instrumental predictive marker has established a compelling avenue for further exploration.

## Methods

### Sample recruitment

Ethical approval for this study was granted by the Institutional Review Board of the the Faculty of Medicine, Chulalongkorn University, Bangkok, Thailand (Med Chula COA No. 0678/2022, IRB no. 1643/2564). The retrospective study included all patients who underwent preoperative therapy at Chulalongkorn University's Division of Plastic and Reconstructive Surgery, Department of Surgery, Faculty of Medicine, Bangkok, Thailand, from 2012 to 2018. Informed consent to participate in the investigation was obtained in writing from all subjects and/or their legal guardian(s) in accordance with the Declaration of Helsinki. Prior to surgery, no patients were administered radiation therapy or chemotherapy. Inadequate pathologic tissue, absence of clinical data, and insufficient follow-up are exclusion criteria.

KR and NK confirmed the diagnosis of cutaneous melanoma and the subtype designation in all patients according to haematoxylin and eosin (H&E)-stained slides. Comprehensive clinical and follow-up data were collected, and the adequacy of the tumour tissue in the formalin-fixed paraffin-embedded tissue blocks was determined. The duration from surgery to recurrence, death, or final follow-up was documented. Ultimately, 48 participants were included in this study. Age, sex, tumour stage, and time of recurrence or death were collected from the clinical data.

### Immunohistochemistry (IHC)

IHC was performed on paraffin-embedded sections of all specimens. Two-micron sections were placed on positively charged slides (SuperFrost Plus; Menzer-Glaser, Freiburg, Germany) and dewaxed with xylene and alcohol. To retrieve antigens, the sections were exposed to Tris–EDTA buffer (pH 9.0) in a microwave at 97 °C for 10 min. Subsequently, a 3.5 N HCl solution was added, and the slides were incubated for 15 min at room temperature. The slides were then subjected to 12 h incubation at 4 °C with a monoclonal antibody against 5-methylcytidine diluted 1:100 (Abcam, Cambridge, UK). A secondary antibody (DAKO EnVision+; DAKO Corp., Santa Clara, California, USA) was then applied, and the samples were incubated at 25 °C for 30 min, followed by visualization using diaminobenzidine chromogen (DAKO Corp.). The positive control was taken from the previous paper on thyroid cancer^12^, which tested positive for 5-mC. The negative control follows the same process but without the antibody against 5-mC.

### Automated digital image analysis

**Figure 3:**
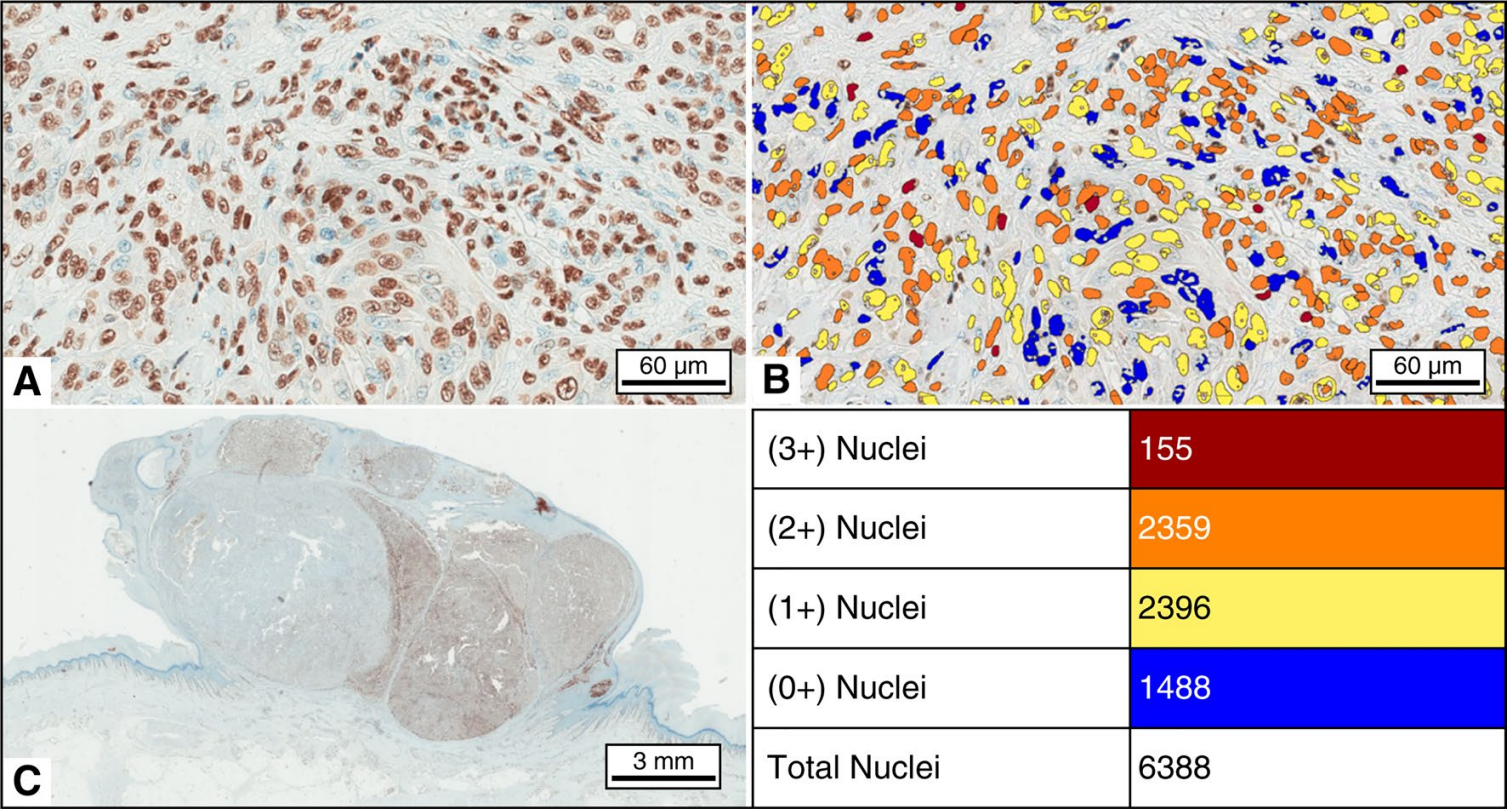
Example of 5-mC staining in melanoma tissue. (**a**) Immunohistochemistry showing nuclear staining at 40 × magnification. (**b**) The immunostaining results in the chosen area were evaluated using the Aperio digital image analysis programme (× 40). The intensity was classified as negative (blue), mildly positive (yellow), moderately positive (orange), or strongly positive (red). The table below shows the count of stained cells. (**c**) The staining pattern observed in melanoma tissue. Based on the figure, the H scores were determined for these slides using the formula: H score = ((155/6398) × 100) × 3 + ((2359/6393) × 100) × 2 + ((2396/6398) × 100) × 1 = 118.5).

All slides stained with the anti-5-methylcytidine antibody were scanned digitally using an Aperio CS2 slide scanner (Leica Biosystems, Wetzlar, Germany). Subsequent image analysis was performed using the Aperio Imagescope v.10.2.2.2352 software package (Aperio Technologies, Irvine, CA, United States). For comparative analyses, both tumour and normal tissue samples from the same patient were examined. To ensure impartiality and reduce selection bias, the regions for examination were arbitrarily selected to cover the superior, inferior, left, right, and central aspects of each image field. A minimum of 5,000 nuclei were evaluated for each patient. The intensity of immunostaining was automatically assessed using the commercially available Nuclear v.9 algorithm (Aperio Technologies, Irvine, CA, United States), and the grading scale was defined in a semiquantitative manner as follows: 0 (negative staining), 1 + (weak nuclear staining), 2 + (moderate staining), and 3 + (strong staining). Simultaneously, the percentage of nuclei with each staining level was determined, as illustrated in Fig. 3. The intensity and proportion scores were averaged to generate a 5-methylcytidine immunohistochemical score, which represented the degree of methylation for each patient and was calculated by the following formula for histochemical scoring (H score) assessment; this score incorporates both the staining intensity and the percentage of stained cells at each intensity level^45,46^.

$$ \frac{{\left( {\% {\text{ of 3}} + {\text{ nuclei }} \times { 3}} \right) \, + \, \left( {\% {\text{ of 2}} + {\text{ nuclei }} \times { 2}} \right) \, + \, \left( {\% {\text{ of 1}} + {\text{ nuclei }} \times { 1}} \right)}}{100} $$

### Statistical analysis

The data were analysed using the SPSS Windows Version 22 programme (SPSS, Inc., Chicago, IL, USA). Utilised Pearson's chi-square test and Fisher's exact test to determine a potential correlation between clinicopathological factors and 5-mC H scores. An analysis of variance (ANOVA) was conducted to compare multiple groups. Generating Kaplan–Meier curves was used to calculate disease-free survival (DFS) and overall survival (OS). The Cox regression model served as the foundation for both the univariate and multivariate survival analyses. p < 0.05 indicated statistical significance.

### Ethics declarations

Ethical approval for this study was granted by the Institutional Review Board of the Faculty of Medicine, Chulalongkorn University, Bangkok, Thailand (Med Chula COA No. 0678/2022, IRB no. 1643/2564). Patients with cutaneous melanoma requiring preoperative therapy between 2012 and 2018 were enrolled from the Division of Plastic and Reconstructive Surgery within the Department of Surgery of the Faculty of Medicine at Chulalongkorn University, Bangkok, Thailand.

## Data Availability

All data generated or analysed during this study are included in this published article.
